# Withstanding the test of time for patients in oncology clinical trials

**DOI:** 10.1093/oncolo/oyag176

**Published:** 2026-05-07

**Authors:** Katherine Zhuo, Rahul Banerjee

**Affiliations:** Department of Medicine, University of Washington, Seattle, WA 98109, United States; Department of Medicine, University of Washington, Seattle, WA 98109, United States; Clinical Research Division, Fred Hutchinson Cancer Center, Seattle, WA 98109, United States

**Keywords:** time toxicity, clinical trials, quality of life

In this issue of *The Oncologist*, Ouimet and colleagues describe the results of their analyses of planned time burden across 73 Phase II clinical trial arms in oncology spanning a wide range of solid tumors and hematologic malignancies.^1^ They find that most patients who participated in these Phase II trials spent a median of 2 days per month on research-related activities (higher during the first month of therapy) and underwent a median of 13 separate blood draws per month. While neither amount is particularly alarming in isolation, the authors then find that planned research days encompassed over 10% of median progression-free survival (PFS) and 4% of overall survival (OS) for participants in these Phase II trials. Their findings draw important scrutiny to clinical trial design in this setting.

For context, relative time burden as a proportion of OS is generally higher in settings where survival is shorter (Table 1).^1–4^ This principle is particularly true with Phase I trials, which are often first-in-human investigations for patients with no remaining treatment options. Even for drugs that ultimately prove to be quite effective, some degree of time burden can be expected in their original Phase I trials given the initial requirement for more intensive safety monitoring and pharmacokinetic assessments.^4–6^ In contrast, Phase II trials generally involve established dosing regimens with slight modifications in dosing details or sequencing permutations. For example, in the current analysis of 73 Phase II trial arms by Ouimet and colleagues,^1^ 92% involved open-label drugs and 41% involved drugs that had already received regulatory approval for other indications. Planned time burden comprised roughly 4% of OS in their analyses regardless of treatment intensity (monotherapy versus combination therapy), route of administration (oral versus intravenous), or cancer stage. Interestingly, the percentage of OS spent on research days was lower in industry-sponsored trials compared to investigator-initiated trials: medians 3.7% versus 5.9% of all days spent alive.^1^

**Table 1.** Selected analyses of time burden in advanced cancers.

Study	Population	Years	Median OS	Median time burden
**Bange 2020** ^2^	Real-world	2011-2019	7.6 mo	10% of OS
**Gupta 2023** ^3^	Phase III trials	2003-2005	6.1 mo	18% of OS
**Iskander 2025** ^4^	Phase I trials	2020-2022	11.9 mo	4% of OS
**Ouimet 2026** ^1^	Phase II trials	2023-2024	11.2 mo	4% of OS

Details of each analysis are omitted for brevity. Median time burden refers to the median proportion of overall survival in days that was spent in clinic or hospital settings, including trial-related activities.

Abbreviations: mo, months; OS, overall survival.

There are of course limitations to the generalizability of the authors’ findings, most notably that the median PFS in the authors’ analysis across all trial arms was only 3.7 months (range: 0.9-27.7 months) and median OS 11.2 months (range: 3.6-48.6 months). These clinical outcomes, while unfortunately the norm with certain malignancies, likely do not reflect time burden in more indolent malignancies such as prostate cancer or multiple myeloma that were underrepresented in the authors’ analysis. It is also difficult to differentiate in hindsight what elements of time burden are attributable to the trial itself versus the basic level of monitoring inherent to living with a given type of cancer. For example, in an analysis of trial participants with non-small cell lung cancer versus matched real-world patients later receiving the same regimen, median healthcare contact days until patient death were 79 days for trial participants versus 68 for standard-of-care patients—in other words, somewhat higher but not dramatically so.^7^

Whether the authors’ reported time burden reported by the authors for Phase II trials rises to the level of time toxicity is impossible to say. While healthcare contact days certainly serve as a metric for time burden, time toxicity more broadly describes the cumulative burden of cancer care that displaces time otherwise spent on usual life activities.^8^ Even within a specific cancer type or regimen, how “time-toxic” a regimen is perceived to be can vary significantly based on patient-specific and visit-specific factors.^9–11^ While one patient may view an extra trial-required visit to be a burden on them and their loved one, a different patient may view it as a healing-oriented encounter to ensure that they feel supported while receiving an investigational regimen. Regardless, there is no doubt that the uniquely high time burden of clinical trial participation can lead to disparities based on overlapping social determinants of health including income, geographic location, and caregiver access.^12–14^ Patients from underserved backgrounds may face greater difficulties accommodating both expected and unexpected time burdens in clinical trials, thereby increasing the risks of non-enrollment or early dropout for these vulnerable populations.^15^ Moreover, the higher time burden observed by the authors with investigator-initiated trials than with industry-sponsored trials—even if partially confounded by academic trials generally enrolling less fit patients or more aggressive disease indications—forces us to point an uncomfortable finger inward at our own practices in clinical trial design.

**Figure 1.**
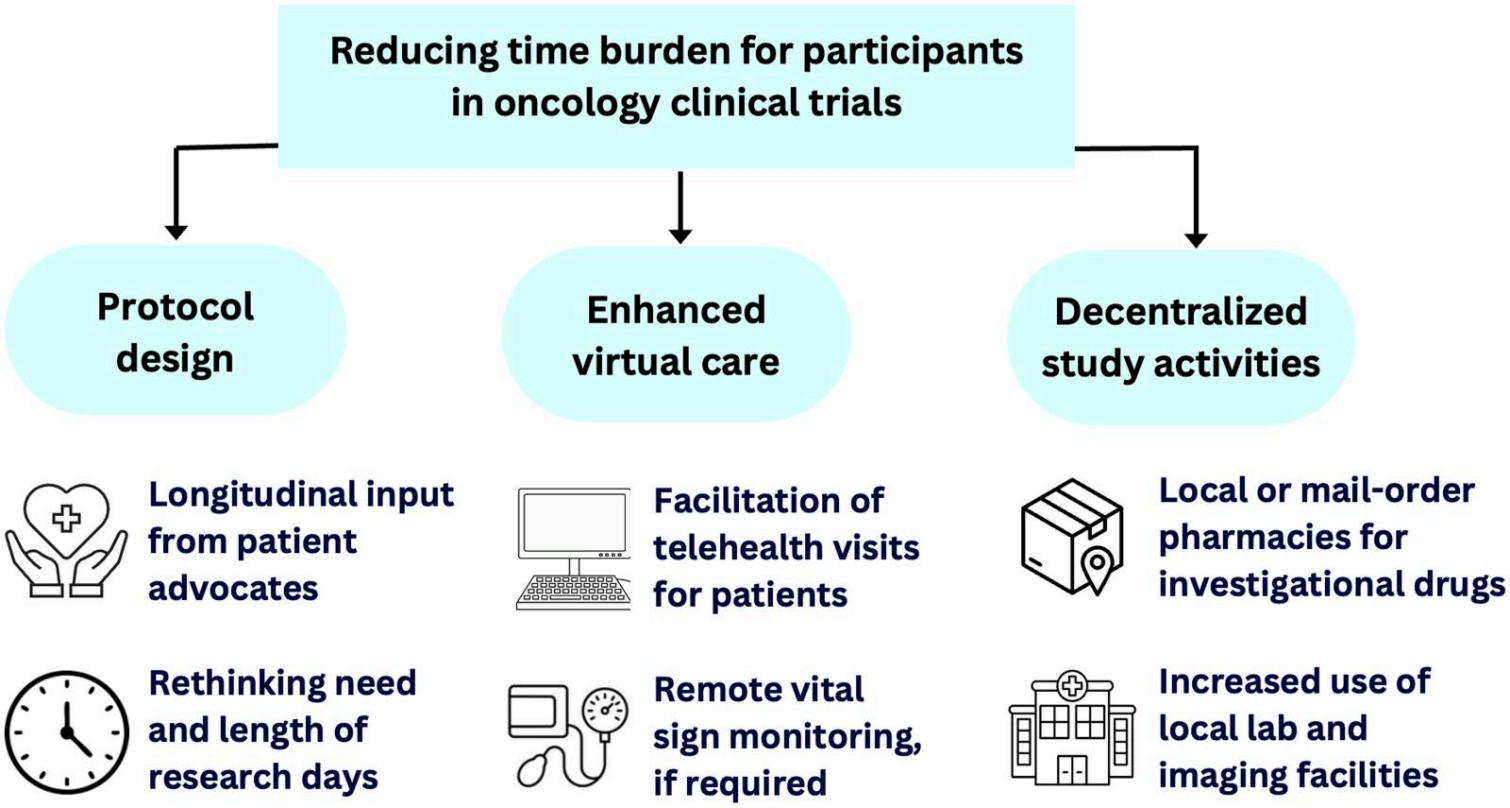
Strategies to lower time burden in clinical trials.

Thankfully, Ouimet and colleagues also describe many potential solutions to reduce the time burden of clinical trials in oncology (Figure 1).^1^ These strategies may also improve the diversity of patient populations captured in clinical trials. As one example, decentralized trial designs to allow for local center-based or even home-based assessments can significantly reduce the disruptiveness of a given trial interaction for individual patients. At its extreme, a “virtual-first” paradigm where telehealth interactions are the norm rather than the exception have been proposed in the field.^16^^,^^17^ As a second example, the authors highlight the importance of patient advocates in clinical trial design to identify activities that are less necessary or more burdensome for participating patients. Importantly, the value of patient advocates applies not just to traditional research-only clinic encounters but also to less visible study-related activities such as patient-reported outcome measurements and general logistics.^18–20^ Ultimately, while there is no perfect “test of time” to completely capture time burden and time toxicity across the lifespan of a patient and a given trial, these important steps offer the potential to improve access to clinical trials and thus the generalizability of the trial results thereafter.
